# Global trends in diabetes-related mortality with regard to lifestyle modifications, risk factors, and affordable management: A preliminary analysis

**DOI:** 10.1016/j.cdtm.2021.03.003

**Published:** 2021-04-15

**Authors:** Nikolai Khaltaev, Svetlana Axelrod

**Affiliations:** a Global Coordination Mechanism on the Prevention and Control of NCDs, Geneva 1208, Switzerland; b High School of Health Administration, First Moscow Medical Academy, Moscow 119991, Russia

**Keywords:** Diabetes, Tobacco, Physical activity, High blood pressure, World Health Organization

## Abstract

**Background:**

According to the World Health Organization (WHO), a global reduction of 17% has been achieved in the major noncommunicable disease-associated mortality rate since 2000. This decline was due to the decreasing mortality associated with cardiovascular and chronic respiratory diseases. The WHO has not made any comments on diabetes-related mortality thus far. The objective of this study was to demonstrate trends in diabetes-related mortality associated with country-wide interventions.

**Methods:**

The WHO statistics were used to assess trends in diabetes-related mortality from 2000 to 2016. Different types of community-based interventions in 49 countries were compared and assessed.

**Results:**

The baseline mortality decreased by 7%. Mortality in middle-income countries was higher than that in high-income countries. The prevalence of obesity showed a gradual increase in all countries. After implementation of the WHO “best buy” in 2010, mortality increased in 17 countries and decreased in 32 countries. Regarding the smoking prevalence trend, 87% countries with decreasing diabetes-related mortality had a gradual decline in tobacco usage since 2000. The decline was observed only in 43% countries with increasing diabetes-related mortality. The prevalence of hypertension increased in 19% countries with declining diabetes-related mortality and in 35% countries with increasing diabetes-related mortality. Physical activity measures tended to be better implemented in countries with declining diabetes-related mortality than in countries with increasing diabetes-related mortality.

**Conclusion:**

Smoking cessation and better blood pressure control are associated with declining diabetes-related mortality. Longer implementation periods are needed for other lifestyle interventions.

## Introduction

Diabetes is categorized as a major noncommunicable disease (NCD), addressed by the World Health Organization (WHO) in the global action plan for the prevention and control of NCD^1^ and the WHO's 13th general program of work 2019–2023.^2^^,^^3^ There are different types of diabetes—type 1 or insulin-dependent diabetes; type 2, non-insulin-dependent, or adult-onset diabetes; and gestational diabetes. Most diabetic patients have type 2 diabetes.^4^ Four hundred and twenty-two million adults globally were living with diabetes in 2014. A total of 1.5 million diabetes patients died in 2012. High blood glucose levels (fasting plasma glucose level ≥7 mmol/L) led to additional 2.2 million deaths caused by the increased risk of cardiovascular diseases (CVD), leg amputation, kidney failure, nerve damage, and vision loss.^5^, ^6^, ^7^ Uncontrolled diabetes increases the risk of fetal death and other complications in pregnancy.^8^ Diabetes and its complications cause huge economic losses to patients and their families, health systems, and national economies.^9^^,^^10^ Type 2 diabetes and its complications are preventable through regular exercise, a healthy diet, smoking cessation, and blood pressure and lipid control. An approach involving the government and the general population is needed for effective prevention of this disease.^5^ Many countries have national diabetes policies as part of their national NCD policy, which along with diabetes, address other major NCDs, cardiovascular diseases (CVDs), cancer, and chronic respiratory diseases (CRDs) that are preventable through public policies that address their common risk factors—tobacco use, excessive alcohol intake, unhealthy diet, physical inactivity, and air pollution.^11^ The action plan of the policy sets targets initially developed by the WHO, ministries of health, and other government departments. Their global targets are aligned with the United Nations 2030 Agenda for Sustainable Development Goals (SDG). The SDG Target 3.4 states to reduce premature mortality from NCDs by one-third by 2030 through prevention and treatment and promote mental health and well-being.^12^^,^^13^

Five years after the adoption of the NCD action plan and 3 years after the formulation of SDG Target 3.4, the WHO's independent high-level commission on NCDs was convened by the WHO Director General to advise him with concrete recommendations on how countries can accelerate their progress toward attaining SDG Target 3.4.^14^ The commission stated that the probability of dying from any one of the four major NCD is declining partly due to the decreasing mortality in only two categories, CVDs and CRDs. The 17% decline in the global rate of NCD-related mortality from 2000 to 2015 is not enough to meet SDG Target 3.4 by 2030.

The commission gives no comments concerning the dynamics of diabetes-related mortality. In this article, we have reported trends in diabetes-related mortality associated with lifestyle changes and affordable management implemented by the WHO at the country level as “best buy” interventions.

## Methods

### Mortality trends

The WHO statistics, based on unified mortality and cause-of-death reports, were used to analyze global NCD mortality trends and compare and assess the different types of community-based and country-wide interventions.^15^ All data received from each country were separated into five categories. Only countries with multiple years of national death registration data and high completeness and quality of cause of death assignments were included in the analysis. Estimates for these countries may be compared and time series may be used for priority setting and policy evaluation.^5^ The E10-E14 criteria were used for diabetes coding according to the International Classification of Diseases (ICD)-10 classification. The WHO attributed diabetes-related mortality caused by CVD to mortality from diseases of the circulatory system. Changes in coding practice from ICD-9 to ICD-10 have been considered to ensure that all countries in the study used the same ICD-10 code throughout the follow-up period. For example, for Mauritius, a change in the coding practice for diabetes was corrected by an adjustment in diabetes-related deaths before 2005 upward by a factor of 3.3. The coding system adjustment has also been mentioned.^1^^,^^15^ Only 49 of 183 countries with multiple years of national death registration data and high completeness and quality of cause-of-death assignments were included in the analysis. Estimates for these countries may be compared, and time series may be used for priority setting and policy evaluation. The preparation of these statistics was undertaken by the WHO Department of Information, Evidence, and Research in collaboration with WHO technical programs. Documentation and regional-level summary tables are available on the WHO website.^15^

### Mortality estimates

Total diabetes-related mortality from 2000 to 2016 with the interim analysis in 2010 and 2015 was analyzed in 49 countries, including 36 high-income countries (HICs; Australia, Austria, the Bahamas, Belgium, Brunei Darussalam, Canada, Chile, Croatia, Czechia, Denmark, Estonia, Finland, France, Germany, Hungary, Iceland, Ireland, Israel, Italy, Japan, Latvia, Lithuania, Luxemburg, Malta, the Netherlands, New Zealand, Norway, the Republic of Korea, Slovakia, Slovenia, Spain, Sweden, Switzerland, Trinidad and Tobago, the United Kingdom, and the United States of America) and 13 middle-income countries (MICs; Armenia, Brazil, Cuba, Grenada, Guatemala, Kyrgyzstan, Mauritius, Mexico, North Macedonia, the Republic of Moldova, Romania, Saint Vincent and the Grenadines, and Uzbekistan) according to the World Bank classification.^16^

### Lifestyle modifications, diabetes management, and risk factors

The WHO identified a package of 16 NCD management and lifestyle “best buy” interventions that are cost-effective, affordable, feasible, and scalable in all settings. The “best buys” were well described and introduced in 2010 and then updated in 2017.^17^^,^^18^ Implementing all 16 “best buys” in all member states between 2018 and 2025 would avoid 9.6 million premature deaths.^11^

Diabetes-attributable *lifestyle* “best buy” interventions include tobacco, unhealthy diet, physical inactivity, and excessive alcohol intake (see attached questionnaires). To quantify lifestyle modification achievements, we gave 2 points for fully achieved activities, 1 point for partially achieved activities, and no points for unachieved activities, no response, or “don't know” responses.

Diabetes management and availability of essential medicines and basic technologies indicators were assessed along with 10 essential NCD medicines (aspirins, angiotensin-converting enzyme inhibitors, statins, long-acting calcium channel blockers, thiazide diuretics, beta-blockers, metformin, insulin, bronchodilators, steroid inhalants) and six basic measurement tools (weighing scales, height measuring equipment, sphygmomanometer, blood sugar and blood cholesterol measurement devices with strips, and urine strips for albumin assay).^19^ Four-fifths of essential NCD medicines are relevant to the management of diabetes and its complications. All basic technologies are relevant to the diagnosis and treatment of diabetes.

The countries reported the number of essential NCD medicines or essential NCD technologies as “generally available,” for example, 10 of 10 and 6 of 6.

### Assessment of obesity, harmful use of alcohol, insufficient physical activity status, and air pollution

Obesity control is not on the “best buy” list, although this risk factor could be an indicator of physical inactivity and unhealthy diets.^20^ Indoor and outdoor air pollution have also been considered a major public health problem associated with major NCDs.^11^

Obesity was defined as the percentage of the population aged ≥18 years having a body mass index (BMI) ≥30 kg/m^2^ in adults and as the percentage of the population aged 10–19 years > 2 standard deviations above the median of the WHO growth reference for children and adolescents in adolescents.

Ambient air pollution was defined as the exceedance of the WHO guideline level for the annual mean concentration of particles measuring ≤2.5 μm in diameter in the air (by a multiple of). Household air pollution was determined by the percentage of the population with primary reliance on polluting fuels and technologies.^11^ Alcohol consumption was assessed as total alcohol per capita consumption in liters of pure alcohol.^11^ Insufficient physical activity was defined as the percentage of population aged ≥18 years who were physically inactive, defined as not meeting the WHO recommendations on physical activity for health.^11^

Adult risk factor trends for 2000, 2005, 2010, and 2015 were registered for tobacco smoking, obesity, and high blood pressure (HBP). Current tobacco smoking was defined as the percentage of the population aged ≥15 years who smoke any of the tobacco products. Raised blood pressure was defined as the percentage of the population aged ≥18 years having systolic blood pressure of ≥140 mm Hg and/or a diastolic blood pressure of ≥90 mm Hg.^11^

### Statistical analysis

All statistical analyses were conducted in SPSS version 22.0 (SPSS Inc., Chicago, IL, USA). Continuous data, expressed as mean ± standard deviation (SD), were analyzed using Student's *t*-test. A two-sided *P* value < 0.05 was considered statistically significant.

## Results

Baseline (year 2000) age-standardized mortality rate in all analyzed countries was 25.3 ± 31.6 per 100,000. It reduced to 23.4 ± 31.0 per 100,000 in 2016 (*n* = 49, *P* > 0.05). Baseline mortality rate in HICs was 17.9 ± 24.0 in 2000, which decreased to 15.3 ± 21.9 per 100,000 in 2016 (*n* = 36, *P* > 0.05). Baseline mortality rate in MICs was 45.6 ± 41.2 in 2000, which was significantly higher than that in HICs (*t* = 2.29, *P* < 0.05). It reduced to 48.1 ± 42.8 in 2016 (*P* > 0.05). The difference in mortality rate between MICs and HICs in 2016 also remained statistically significant (*t* = 2.64, *P* < 0.02). To better demonstrate the dynamics of diabetes-related mortality and a high diversity of the mean mortality indicators, we analyzed the percentage of mortality changes with respect to the baseline level. On average, by 2016, the total diabetes-related mortality declined by 7% in 49 countries (6.9 ± 34.2). In HICs, it declined by 12% (12.5 ± 32.4), while in MICs, it increased by 11% (10.7 ± 32.9). To demonstrate the dynamics of diabetes-related mortality, we selected four countries with the most visible decline in mortality and four countries with the most visible increase in the same parameter.

Age-standardized death rates and trends in countries with more than 40% decline (Slovenia 59%, Republic of Korea 54%, Switzerland 42%, and the Netherlands 42%) and more than 60% increase (Guatemala 89%, Czech Republic 82%, Latvia 68%, and Austria 63%) are presented in Table 1. All these countries were HICs, except Guatemala, which is a lower MIC. Reviewing smoking prevalence trends from 2000 to 2015,^1^ we found no data for Guatemala and a visible decline in Slovenia for both males and females. The trend in HBP remained unchanged in both countries, while that in obesity prevalence increased in both countries. We observed a significant difference in household levels of air pollution, which was 11 times higher in Guatemala than in Slovenia, with 55% and 5% of the population relying primarily on polluting fuels and technologies in Guatemala and Slovenia, respectively.^11^ Regarding NCD management, at least 50% of health facilities utilized CVD management guidelines in Slovenia, while this level of coverage could not be attained in Guatemala. We also found a clear difference in the availability of medicines and technologies between Slovenia (100%) and Guatemala, with only 60% (6 of 10) for medicines and 50% (3 of 6) for technologies. We observed trends in the opposite direction when analyzing mortality in Slovenia declining and Austria increasing, which are neighboring European Union countries. Slovenia underwent dramatic changes in its political system and socioeconomic conditions after the collapse of Yugoslavia, while Austria remained stable in these aspects. Concerning CVD in these countries, Slovenia reported having CVD guidelines that are utilized in at least 50% of health facilities in 2017, while Austria could not report of having the same guidelines. The prevalence of diabetes in adults was 10% in Slovenia and 6% in Austria in 2014. The prevalence of tobacco use in adults was 27% in Austria and 20% in Slovenia in 2016. Trends in tobacco use from 2000 to 2015 for each country demonstrate a decline in both countries.^11^ Diabetes coding practice has been well adjusted in both countries. Therefore, despite the fact that these countries are neighbors, the dynamics of diabetes-related mortality are very different, with socioeconomic factors playing a key role in this case.

**Table 1:** Age-standardized diabetes-related mortality rate per 100,000 persons in selected countries with different mortality trends, both sexes, 2000–2016.

Countries	Decreased/increased rate (%)	Mortality rate with years (%)			
		2000	2010	2015	2016
*Mortality decreased*					
Slovenia	59.0	16.1	6.1	6.8	6.6
The Republic of Korea	54.0	29.9	18.1	14.2	13.9
Switzerland	42.0	12.8	9.1	7.4	7.4
The Netherlands	42.0	10.6	6.7	6.2	6.1
*Mortality increased*					
Guatemala	89.0	36.2	63.9	67.0	68.3
The Czech Republic	82.0	8.7	9.8	16.0	15.8
Latvia	68.0	6.5	13.2	10.5	10.9
Austria	63.0	8.7	14.9	15.2	14.2

To further analyze the association of mortality dynamics with “best buy” implementation and the level of NCD risk factors in all 49 countries, we compared 17 countries (35% of which were MICs) where diabetes-related mortality increased after 2010 and 32 countries (22% of which were MICs) where diabetes-related mortality decreased. No significant difference in lifestyle modification measures was found between the groups (Table 2). However, physical activity measures had a strong tendency to be better implemented in countries with declining diabetes-related mortality than in countries with increasing diabetes-related mortality (*t* = 1.85, *P* < 0.1).

**Table 2:** Lifestyle modification measures in countries with different diabetes-related mortality trends from 2010 to 2016.

Lifestyle modifications	Declined countries scores (*n* = 32, mean ± SD)	Increased countries score (*n* = 17, mean ± SD)	*T*	*P*
Tobacco demand reduction measures	5.5 ± 2.3	4.8 ± 2.2	1.03	<0.05
Harmful use of alcohol reduction measures	2.7 ± 1.3	2.6 ± 1.1	0.29	>0.05
Unhealthy diet reduction measures	2.5 ± 1.6	2.1 ± 1.6	1.12	>0.05
Public education and awareness campaign on physical activity	1.8 ± 0.7	1.3 ± 1.0	1.85	<0.1

We found no significant difference between groups at the level of NCD risk factors (Table 3). However, we observed a clear tendency toward a higher level of air pollution, both ambient and household, in countries with increasing diabetes-related mortality. The obesity status was approximately the same in both groups of countries.

**Table 3:** Mean population levels of NCD risk factors in countries with different dynamics of diabetes-related mortality by 2016.

Risk factors	Mortality declined countries, *n* = 32	Mortality increased countries, *n* = 17	*P*
smoking in people aged ≥15 years (%)			
Males	30.1 ± 10.3^a^	31.6 ± 10.6^c^	>0.05
Females	15.8 ± 7.4^a^	16.8 ± 9.1^c^	
Total	22.7 ± 6.0^a^	23.9 ± 7.4^c^	
Physical inactivity in people aged ≥18 years (%)			
Males	29.6 ± 6.2^a^	24.2 ± 10.7^c^	>0.05
Females	38.3 ± 8.7^b^	33.7 ± 11.2^c^	
Total	34.1 ± 7.1^b^	30.00 ± 9.8^c^	
Obesity in people aged ≥18 years (%)			
Males	21.8 ± 7.2	23.4 ± 6.9	>0.05
Females	24.4 ± 7.2	26.2 ± 5.5	
Total	23.1 ± 6.5	24.7 ± 5.8	
Harmful use of alcohol^d^			
Males	14.9 ± 5.0	15.9 ± 5.9	>0.05
Females	3.6 ± 1.6	4.0 ± 1.8	
Total	9.1 ± 3.2	9.6 ± 3.8	
Raised blood pressure^e^ (%)			
Males	28.0 ± 5.9	30.4 ± 8.5	>0.05
Females	22.6 ± 5.4	24.3 ± 7.1	
Total	25.2 ± 5.2	27.2 ± 7.5	
Ambient air pollution^f^	1.1 ± 0.8	1.6 ± 0.9	<0.1
Household air pollution^g^ (%)	5.9 ± 3.2	11.5 ± 13.5	<0.1

a *n* = 30.

b *n* = 31.

c *n* = 14. Data are presented as mean ± SD.

d Total alcohol per capita consumption in liters of pure alcohol.

e The percentage of the population aged ≥18 years with systolic blood pressure ≥140 mm Hg and/or diastolic blood pressure ≥90 mm Hg.

f Ambient air pollution was defined as the exceedance of the WHO guideline level for the annual mean concentration of particles measuring ≤2.5 μm in diameter in the air (by a multiple of).

g Household air pollution was determined by the percentage of the population with primary reliance on polluting fuels and technologies.

**Fig. 1:**
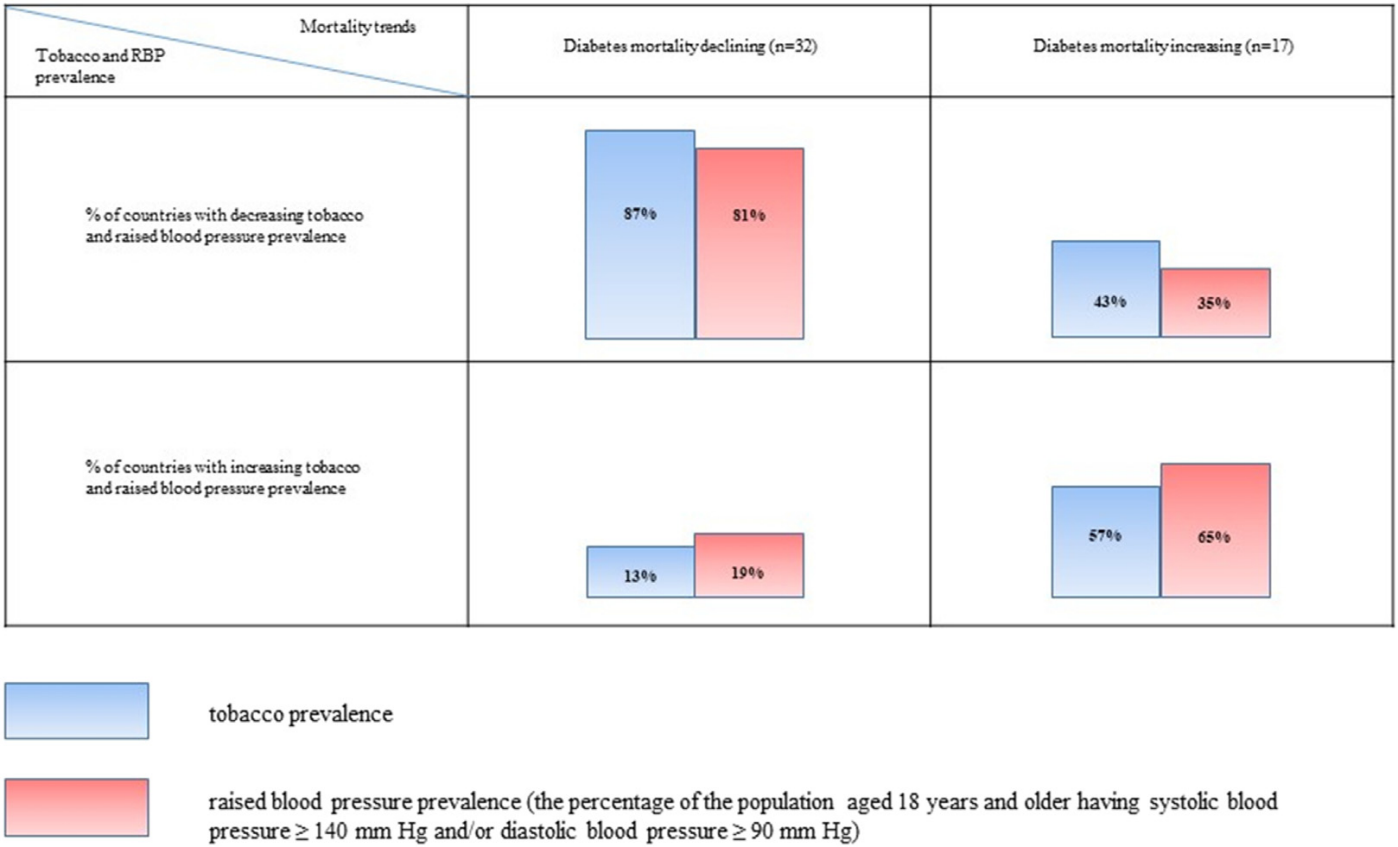
Tobacco and raised blood pressure (RBP) prevalence dynamics (2000–2015) in countries with different diabetes-related mortality trends (2010–2016).

Although we did not find a significant difference in the current prevalence of smoking between countries, the smoking prevalence trends from 2000 to 2015 showed that 87% countries with decreasing diabetes-related mortality had a decline in tobacco usage.^11^ However, only in 43% countries with increasing diabetes-related mortality had a decline in tobacco usage. The prevalence of HBP during the same period, 2000–2015, increased only in 19% countries with declining diabetes-related mortality, while it was increased in 35% of the countries with increasing diabetes-related mortality (Fig. 1).

The rate of availability of essential NCD drugs was 91% in countries with declining mortality and 76% in countries with increasing mortality. A similar trend was observed for the rate of availability of essential NCD technologies, with 81% and 71% for countries with declining and increasing mortality, respectively.

Obesity trends were gradually increasing in both group of countries since 2000.^11^

## Discussion

The mean values of diabetes-related mortality in our study were lower in HICs than in MICs, and this difference did not change over the 16 years of observation. When we analyzed the percentages of changes, we observed a 12% decline in the mortality rate in HICs and an 11% increase in the mortality rate in MICs. This discrepancy corresponds to the WHO trends on the prevalence of diabetes by country income group from 1980 to 2014, in which the highest increases in diabetes prevalence occurred in low MICs (LMICs).^7^ It means that increasing diabetes prevalence is associated with the higher mortality seen in our MICs. This could be a result of better control of diabetes in HICs. Although we found no significant difference in obesity prevalence or dietary modification measures in countries with different dynamics of diabetes-related mortality, these risk factor modifications have a great potential for diabetes prevention. According to various estimates, excess weight and obesity are closely associated with type 2 diabetes and are the driving force for the observed epidemics of type 2 diabetes. The NCD Risk Factor Collaboration Group have demonstrated the growing obesity trends from 1975 to 2014 in 186 countries and concluded that if the post-2000 dynamics continue, the probability of meeting the global target of halting the increase in the prevalence of obesity by 2025 would be <1%.^21^

According to the Institute for Health Matrices and Evaluation, currently, 2.1 billion people are either obese or overweight. Between 1980 and 2015, the worldwide prevalence of obesity has increased by two-fold and diabetes by four-fold in 70 countries and has continuously increased in many other countries.^22^ Obesity is in the center of the metabolic syndrome, which includes elevated blood pressure, high blood sugar, excess body fat around the waist, abnormal lipid spectrum, and a high risk of CVD and diabetes.^23^ The dynamics of diabetes-related mortality in our study, either positive or negative, were observed along with a gradually growing prevalence of obesity in all countries since 2000. The negative dynamics could be due to the influence of other factors such as previous long–term activities in tobacco control, diet and physical activity modification measures, or diabetes and associated CVD management. A 6-year intervention with lifestyle changes in China resulted in the reduction of the incidence of diabetes by 33% in the diet-alone group and by 47% in the exercise group compared with the control group.^24^

The Finnish Diabetes Prevention Study using lifestyle intervention achieved a 58% reduction in diabetes risk.^25^ The US Diabetes Prevention Program has demonstrated a reduced incidence of diabetes in people at high risk after 2.8 years of weight reduction and at least 150 minutes of physical activity per week. This intervention was more effective than treatment with metformin.^26^ Although we did not observe differences in dietary measures (probably due to the fairly short period of intervention), physical activity measures associated with diabetes-related mortality are encouraging (Table 3).

Smoking increases the risk of all diabetes-related health problems, leading to insulin resistance and chronic inflammation, which can accelerate macrovascular and microvascular complications.^27^^,^^28^ Eighty-eight prospective studies with 5,898,795 participants and 295,446 incidental cases of type 2 diabetes demonstrated a dose–response relationship between current smoking and the risk of diabetes. The most visible correlation was seen in active smokers. It was estimated that 11.7% cases of type 2 diabetes in men and 2.4% cases in women were attributable to active smoking. In new smoking quitters, the risk of diabetes is increased (probably due to increasing body mass after quitting); however, as gradually over a period of time, it substantially decreases.^29^^,^^30^ Global activities against tobacco led to a decrease in the global prevalence of tobacco smoking from 27% in 2000 to 20% in 2016.^1^ Our analysis shows that 15 years of declining tobacco usage contribute to the decreasing diabetes-related mortality. There are now more former smokers than smokers worldwide. This explains why we did not see a difference between current prevalence of smoking in countries with different diabetes-related mortality. However, the analysis of smoking trends from 2000 to 2015 has clearly demonstrated a higher diabetes-related mortality rate in countries with a less visible decline in tobacco usage.

The association of diabetes-related mortality with air pollution is interesting since air pollution affects many organs in the body, causing or contributing to many illnesses.^31^^,^^32^ Heavy air pollution in China was associated with increased risk of coronary heart disease and stroke.^33^^,^^34^ Comparable findings suggested that clean air actions not only greatly improved air quality but also significantly reduced the burden of these diseases.^35^ This could also explain the diabetes-related mortality trends we observed; the most dramatic growth of diabetes-related mortality was observed in Guatemala, which was associated with a high level of household air pollution. While analyzing the physical inactivity policy, we observe a tendency toward a better achievement of public education and awareness campaigns on physical activity in countries with declining diabetes-related mortality than in countries with increasing diabetes-related mortality. Better management of diabetes could also explain the differences in mortality trends since early diagnosis leads to better treatment and better health outcomes. Access to blood glucose level, tests should be facilitated at the primary healthcare level. Similar concerns also apply to the working systems for referral and counter-referral since patients need regular specialist assessment of diabetes control and treatment for complications. The availability of essential drugs and technologies and better control of HBP in our study were associated with lower diabetes-related mortality. Proper diabetes management corresponds to the conception of integrated NCD management, where diabetes and CVD management can be combined. In HICs with better access to care, including essential technologies and cost-effective treatments, there is a lower mortality rate, while the cost of treatments and access to care remain major challenges in LMICs. The early identification and treatment of diabetes is a potential life-saving strategy. As this approach is a privilege of HICs, much cheaper and more accessible methods are needed for LMICs. Since overweight and obesity control are a big challenge in the analyzed countries, smoking cessation and prevention smoking commencement, along with diet, physical activity, air pollution control, and adequate management, are the key elements in the current strategy against diabetes to achieve the SDG Target 3.4.

The practical outcome of our observation could intensify national efforts. A future evaluation with more countries included in the analysis and an intensive and well-oriented promotional campaign based on the lessons learned will enable the achievement of better results and movement toward the attainment of the SDG Target 3.4.

## Conflict of interest

None.
