# Supplementary material for: Global trends in diabetes-related mortality with regard to lifestyle modifications, risk factors, and affordable management: A preliminary analysis
Source: Chronic Dis Transl Med. 2021 Apr 15;7(3):182–9. doi: 10.1016/j.cdtm.2021.03.003 (PMC8413119; doi:10.1016/j.cdtm.2021.03.003)
Supplement: Supplementary file 1 [file mmc1.zip › cdtm_216_Attachment_mmc1.odt]

Attachement.

 Life style modification questionnaires.

Tobacco demand reduction measures

Implementation by a country of the 5 demand-reduction measures :(1) reduction of affordability by increasing excise taxes and prices of tobacco products; (2) elimination of exposure to second-hand tobacco smoke in all indoor workplaces, public places and public transport; (3) implementation of plain or standardized packaging or large graphic health warnings on all tobacco packages; (4) enaction and enforcement of comprehensive bans on tobacco advertising, promotion and sponsorship; (5) implementation of effective mass media campaigns that educate the public about the harms of smoking, tobacco use and second-hand smoke. Country can obtain maximum 10 points in case of full achievement of all 5 demand-reduction measures.

Harmful use of alcohol reduction measures

Country has implemented, the following three measures to reduce the use of alcohol as per the WHO Global Strategy to Reduce the Harmful Use of Alcohol: (1) enaction and enforcement of restrictions on the physical availability of retailed alcohol (via reduced hours of sale); (2) advertising bans (across multiple types of media), or comprehensive restrictions; (3) increase in excise taxes on alcoholic beverage. Country can obtain maximum 6 points in case of full achievement of all 3 demand reduction measures.

Unhealthy diet reduction measures

Country has implemented the following measures to reduce unhealthy diets: (1) adoption of national policies to reduce population salt (sodium) consumption; (2) adoption of national policies that limit saturated fatty acids and virtually eliminate industrially produced trans fatty acids in the food supply. Country can obtain maximum 4 points in case of full achievement of 2 demand-reduction measures.

Public education and awareness campaign on physical activity

Country has implemented at least one recent national public awareness and motivational communication for physical activity, including mass media campaigns for physical activity behavioral change. Country can obtain maximum 2 points in case of full achievement of this demand-reduction measure.
